# Bleomycin‐Elektrosklerotherapie bei kaposiformem Hämangioendotheliom mit Kasabach‐Merritt‐Phänomen im Erwachsenenalter

**DOI:** 10.1111/ddg.15951_g

**Published:** 2026-04-08

**Authors:** Jakob Veeser, Raphael Wilhelm, Janina Dietzel, Yalda Ghoreishi, Nora Laubach, Nora Elspaß, Maike Kaufhold, Beate Weidenthaler‐Barth, Christian Rose, Stephan Grabbe, Henner Stege, Hadrian Nassabi

**Affiliations:** ^1^ Hautklinik Universitätsmedizin der Johannes Gutenberg Universität, Mainz; ^2^ Dermatohistologisches Labor Bartsch und Rose, Lübeck

Sehr geehrte Herausgeber,

Das kaposiforme Hämangioendotheliom (KHE) ist ein seltener Tumor, welcher vorwiegend im Kindes‐ und Jugendalter auftritt.^1^ Dennoch sind auch einzelne Fälle im Erwachsenenalter beschrieben.^2^, ^3^ Das kaposiforme Hämangioendotheliom ist häufig mit dem Kasabach‐Merritt‐Phänomen (KMP) assoziiert, einer schweren Koagulopathie, die zu einer disseminierten intravasalen Gerinnung (DIC) und einem erhöhten Blutungsrisiko führt. Dieser Bericht beschreibt den Fall eines kaposiformen Hämangioendothelioms (KHE) mit Kasabach‐Merritt‐Phänomen (KMP) bei einer erwachsenen Patientin und beleuchtet die klinischen und histopathologischen Befunde, die diagnostischen Herausforderungen sowie die therapeutischen Ansätze bei dieser seltenen Erkrankung.

Eine 33‐jährige Patientin philippinischer Herkunft stellte sich mit anhaltenden Blutungen aus einem exophytischen, ulzerierten Tumor am rechten Oberarm vor, welcher sich über das vergangene Jahr hinweg entwickelt hatte. Die Patientin berichtete über eine im Vorjahr in ihrem Heimatland gestellte Diagnose eines Angiosarkoms der linken Brust mit anschließender Mastektomie. Weitere Vorerkrankungen bestanden nicht, die Familienanamnese war unauffällig.

Klinisch zeigte sich ein stark blutender Tumor am rechten Oberarm sowie mehrere subkutane, vaskularisierte Knoten am Rumpf und an den oberen Extremitäten (Abbildung 1a, b). Die Patientin war in gutem Allgemein‐ und adipösem Ernährungszustand. Es bestand weder ein einseitiges Längenwachstum einer Extremität noch ein Gigantismus.

**ABBILDUNG 1:**
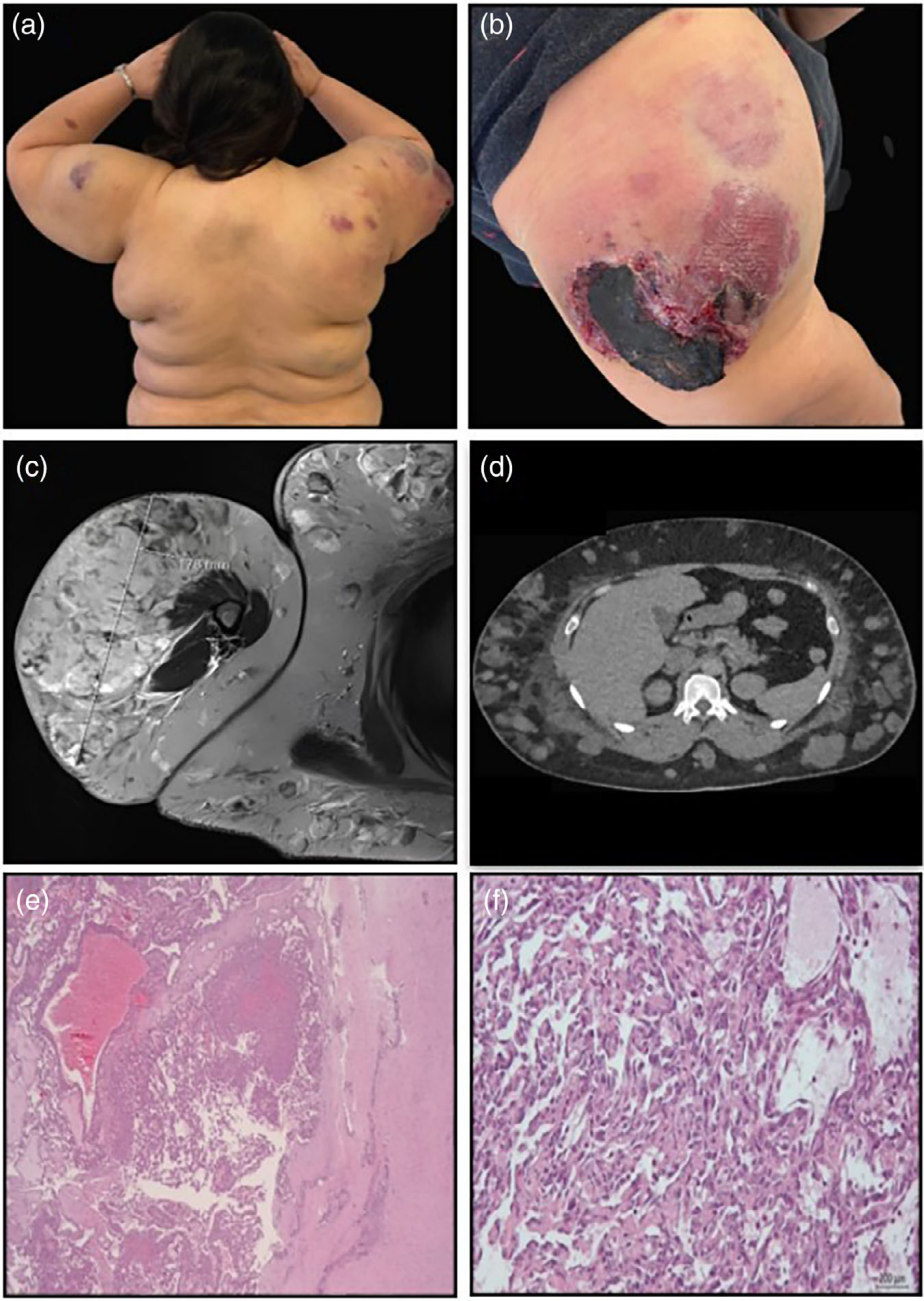
Klinisches, radiologisches und histopathologisches Erscheinungsbild. (a) Multiple subkutane Knötchen; (b) Tumor am rechten Oberarm; (c) MRT des Tumors am Oberarm; (d) CT der subkutanen Knötchen am Rumpf; (e) Histopathologie: × 25; (f) Histopathologie: × 200.

Die Magnetresonanztomographie zeigte eine gemischt‐vaskuläre Raumforderung von etwa 15 × 10 × 18 cm am rechten Oberarm sowie mehrere disseminierte subkutane Raumforderungen am gesamten Integument (Abbildung 1c). In der kontrastmittelverstärkten Computertomographie konnte die Arteria circumflexa humeri posterior als das den Tumor hauptsächlich versorgende Gefäß identifiziert werden (Abbildung 1d).

Die Labordiagnostik ergab eine Thrombozytopenie, eine mikrozytäre Anämie mit einem Hämoglobinwert von 6,8 g/dl sowie verminderte Fibrinogen‐ und Quick‐Werte bei gleichzeitig erhöhten D‐Dimeren. Somit waren die diagnostischen Kriterien einer DIC erfüllt.

Es erfolgte eine genetische Untersuchung der Lymphozyten aus EDTA‐Blut sowie kultivierter Fibroblasten aus einer Biopsie, die aus Hautgewebe angrenzend an eine der subkutanen Raumforderungen entnommen worden war. Diese ergab keinen Hinweis auf Genveränderungen, die mit bekannten genetischen Syndromen mit vaskulären Malformationen assoziiert sind.

Die histologische Untersuchung zeigte zahlreiche spaltförmige Gefäßräume in der gesamten Dermis mit leicht vergrößerten Endothelzellen (Abbildung 1e). Die subkutanen Knoten bestanden aus dicht gepackten, unregelmäßig geformten Kapillaren mit erhöhter Pleomorphie, zytologischer Atypie und einigen Mitosen (Abbildung 1f). Es fanden sich solide Bereiche von spindelförmigen Zellen, die an ein Kaposi‐Sarkom erinnerten. Das umgebende lymphozytäre Infiltrat enthielt nur wenige Plasmazellen. Die immunhistochemische Analyse zeigte eine starke Positivität für CD31, CD34 und ERG in den vaskulären Endothelzellen der Dermis und Subkutis, mit einem umgebenden SMA‐positiven Perizytenmantel. Die Proliferationsrate war in den tieferen Arealen mit bis zu 30 % Ki67‐Positivität erhöht, was auf eine deutliche angiogene Aktivität hinweist. Die Untersuchung auf HHV8 war negativ. Die proliferierenden Gefäßstrukturen in Dermis und Subkutis zeigten überwiegend keine Positivität für Podoplanin. Nach Rücksprache mit einem Referenzpathologen wurden die Befunde als vereinbar mit einem KHE gewertet.

Das kaposiformen Hämangioendotheliom tritt am häufigsten an den oberen Extremitäten, gefolgt vom Rumpf, auf und hat eine geschätzte Prävalenz von 0,91 pro 100 000.^5^ Trotz gelegentlicher familiärer Häufung ist bisher keine genetische Ursache bekannt. Die Erkrankung betrifft vorwiegend Kinder und Jugendliche, es gibt jedoch Einzelfallberichte im Erwachsenenalter.^2^, ^3^ Aufgrund der Seltenheit der Erkrankung in dieser Altersgruppe existieren keine etablierten Therapieprotokolle.

Die initiale Behandlung unserer Patientin konzentrierte sich auf die Partikelembolisation der zuführenden Gefäße des Tumors am rechten Oberarm. Wie von Zhou et al. gezeigt, ist dies eine effektive Methode zur Verbesserung des Kasabach‐Merritt‐Phänomens, insbesondere bei ausgedehnten Läsionen, bei denen eine primäre Exzision aufgrund des Blutungsrisikos nicht möglich ist.^6^

Trotz intensiver Therapieversuche hielt die Blutung an. Eine Woche später wurde daher eine innovative lokale Behandlung, die Bleomycin‐Elektrosklerotherapie (BEST), durchgeführt. Dabei wird zunächst Bleomycin appliziert, gefolgt von mehreren kurzen elektrischen Impulsen, die über feine Nadeln verabreicht werden. Dieser Prozess erhöht vorübergehend die Permeabilität der Zellmembranen für den sklerosierenden Wirkstoff, was dessen intrazelluläre Konzentration im Zielgewebe verhöht. Aufgrund der örtlichen Selektivität und der geringeren Körperdosis sind systemische Nebenwirkungen reduziert.^7^

Unter Allgemeinanästhesie wurden 30 mg Bleomycin (körperoberflächenbasiert berechnet) gelöst in 100 mL NaCl in den Tumor am rechten Oberarm injiziert, wobei die Lösung gleichmäßig verteilt wurde. Anschließend wurden 57 elektrische Impulse mit über 14,5 Ampere mittels einer hexagonalen Elektrode in das Tumorgewebe eingebracht. Abschließend wurde ein Kompressionsverband angelegt.

Als erste systemische Therapie erhielt die Patientin Prednison 60 mg/d (1 mg/kg KG), Vincristin 2 mg/d und Lenalidomid 10 mg/d.^8^

Trotz der einmonatigen Behandlung zeigten sich die disseminierten subkutanen KHE‐Läsionen am Oberkörper weiter progredient, sodass die Therapie auf Sirolimus 3 mg/Tag umgestellt wurde. Dieser Wechsel führte zu einer Verbesserung der DIC, was schließlich die Exzision des großen Tumors am Oberarm ermöglichte.^6^, ^9^ Ein CT‐Staging nach 6 Monaten zeigte eine moderate Regression der verbliebenen kleineren KHE‐Läsionen am Integument.

Dieser Fall unterstreicht die diagnostischen und therapeutischen Herausforderungen des Kasabach‐Merritt‐Phänomens im Erwachsenenalter und stellt mit der Bleomycin‐Elektrosklerotherapie einen möglichen innovativen lokalen Therapieansatz vor. Besonders zur Blutungskontrolle und Erstbehandlung ausgedehnter, nicht primär operabler Läsionen kann dieses Verfahren eine wertvolle Ergänzung zur systemischen Therapie darstellen.

## DANKSAGUNG

Open access Veröffentlichung ermöglicht und organisiert durch Projekt DEAL.

## INTERESSENKONFLIKT

Keiner.
